# Duality of P2X7 Receptor in Amyotrophic Lateral Sclerosis

**DOI:** 10.3389/fphar.2020.01148

**Published:** 2020-07-24

**Authors:** Cinzia Volonté, Susanna Amadio, Francesco Liguori, Paola Fabbrizio

**Affiliations:** ^1^ CNR-Institute for Systems Analysis and Computer Science, Rome, Italy; ^2^ Fondazione Santa Lucia, IRCCS, Rome, Italy; ^3^ Istituto di Ricerche Farmacologiche Mario Negri, IRCCS, Milan, Italy

**Keywords:** amyotrophic lateral sclerosis, drug discovery, P2X7, purinergic, SOD1-G93A mouse

## Introduction

Much has been explained to date about the multifactorial nature of amyotrophic lateral sclerosis (ALS) and about the multiple cellular/molecular targets and genes involved in the disease. Even more has been said about the pleiotropic and sometimes opposite functions of purinergic ionotropic P2X7 receptor. Bearing this in mind, the first question we ask is: why should we tell something more about P2X7 in ALS? The answer is simple: despite some apparently conflicting results, our general understanding supports the fact that the pathological mechanisms of ALS indeed proceed through pathways in which P2X7 plays a crucial and dual role. In our opinion, this topic is surely worth updating and discussing.

Sharing basic research results with clinicians and communicating clinical findings to basic researchers is a chief goal in the efforts to improve human health. Translating basic research to clinic is also a primary aim in the faith to defeat ALS. By building upon some recent success and exciting new pharmacological developments about P2X7 (De Marchi et al., 2016; Rech et al., 2016; Park and Kim, 2017; Pevarello et al., 2017; Górecki, 2019), here we discuss the reasons and the key challenges of fostering research in the field of P2X7 and ALS. In other words, the power of experimental research about a crucial player of inflammation, the P2X7 (Di Virgilio, 2007; Di Virgilio et al., 2017; Di Virgilio et al., 2018), will be exploited to provide further insights in the context of ALS.

ALS is at least two centuries old and is a rare, relentless, multi-layered and heterogeneous familial/sporadic disease targeting motor neurons and additional cell phenotypes as muscles, glia and immune cells (Casterton et al., 2020; Yerbury et al., 2020). It typically causes death within 3–5 years from onset, but it still has no cure because all efforts in the search for treatments have failed so far (Mejzini et al., 2019; Chiò et al., 2020). Current therapies can only reduce morbidity. At present, only two certified FDA drugs exist, the anti-glutamatergic riluzole (Rilutek^®^, Teglutik^®^, approved in 1995) and the free radical scavenger edaravone (Radicava^®^, Radicut^®^, approved in Japan, South Korea, USA, Canada, Switzerland, and China in the years 2015–2019). None of these are fully satisfactory, riluzole having modest benefits on survival of patients and edaravone halting ALS progression only during the early stages (Jaiswal, 2019). To date, more than 50 drugs have failed in ALS clinical trials, while several compounds are currently in interventional phase-III trials (Andrews et al., 2019; Wobst et al., 2020).

Among the most promising:

- Arimoclomol is a hydroxylamine derivative working as a co-inducer of the heat shock proteins and extending the lifespan of superoxide dismutase (SOD)1-G93A mice (the best characterized animal model for ALS) when provided at symptomatic phase (Kalmar et al., 2008).
- Tauroursodeoxycholic acid, a bile acid derivative, is a potent anti-apoptotic agent that preserves motor neurons function by stabilizing the mitochondrial membrane, inhibiting the activation of matrix metallopeptidase 9, and nitrite production (Vaz et al., 2015).
- Methylcobalamin is a vitamin B12 analog that enhances the survival of ALS motor neurons *in vitro* (Ito et al., 2017) and may prolong survival and delay disease progression in patients, if started early (Kaji et al., 2019).
- Masitinib, a type-3 tyrosine kinase inhibitor, is known for modulating neuroinflammatory features, prolonging post-paralysis survival, decreasing aberrant gliosis and motor neuron pathology in the spinal cord of SOD1-G93A rats (Trias et al., 2016).
- Cannabidiol is one of the more than 100 pharmacologically bioactive cannabinoids that have neuroprotective activities by delaying disease progression, motor impairment and prolonging survival in ALS animal models (Urbi et al., 2019).

Despite these encouraging results, novel therapeutic strategies are yet necessary for inspiring further studies and, most importantly, formulating effective treatments (Okano et al., 2020). Thus, the focal question becomes why we should consider a P2X7-targeted strategy for ALS.

The studies about the involvement of purinergic signaling in neurodegenerative and neuroinflammatory conditions are certainly convincing (Volonté et al., 2003; Franke and Illes, 2006; Burnstock, 2008; Khakh and North, 2012; Sperlágh and Illes, 2014; Tewari and Seth, 2015; Burnstock, 2016; Burnstock, 2017a; Burnstock, 2017b) and now flourishing also on ALS and P2X7, in particular (Volonté et al., 2011; Volonté et al., 2012; Volonté et al., 2016; Sebastião et al., 2018; Cieślak et al., 2019; Ruiz-Ruiz et al., 2020). Moreover, because of the evolution of always more specific and potent P2X7 antagonists with a focus on CNS indications (Rech et al., 2016; Pevarello et al., 2017), we can optimistically expect that in the near future some new generation P2X7 drugs might be listed on the formulary and medication plan for ALS patients. In the next sections, we will briefly circumstantiate this belief.

## Central and Peripheral Mechanisms of P2X7 in ALS

Receptors should be more properly analyzed within the cellular context and microenvironment in which they are embedded. Increased immunoreactivity for P2X7 is extensively documented in microglia/macrophages from the spinal cord and brain tissues of ALS patients (Yiangou et al., 2006) and SOD1-G93A mice (D’Ambrosi et al., 2009). Conversely, P2X7 down-regulation is observed in peripheral monocytes of ALS patients (Liu et al., 2016). Depending on the specific cellular context, microenvironment, network of molecules responsible for triggering, maintaining, and terminating the purinergic signaling, i.e. the “purinome” (Volonté et al., 2006; Volonté et al., 2008; Volonté and D’Ambrosi, 2009), we might expect very heterogeneous or even divergent cellular responses triggered by P2X7 itself.

Some interesting concepts about P2X7 become evident, for instance, when we dissect the pathogenic mechanisms of ALS based on the central *versus* peripheral localization of the receptor. Indeed, activation of P2X7 by agonists in ALS primary microglia further aggravates pro-inflammatory responses, as NADPH oxidase 2 activity, reactive oxygen species production, tumor necrosis factor-*α* and cyclooxigenase-2 expression, microtubules associated protein kinases activation, miR-155, miR-125b, miR-146b up-regulation, and miR-22 down regulation, moreover causing direct toxicity towards ALS motor neuron cells (D’Ambrosi et al., 2009; Apolloni et al., 2013b; Parisi et al., 2013; Apolloni et al., 2014; Parisi et al., 2016). Likewise, repeated stimulations of P2X7 cause SOD1-G93A astrocytes in culture to become neurotoxic (Gandelman et al., 2010) toward motor neurons, which are *per se* sensitive to P2X7-induced toxicity by activation of the 90-kDa heat-shock protein/Fas pathway (Franco et al., 2013; Gandelman et al., 2013). Contrary to these deleterious effects, activation of P2X7 by the potent agonist 2′(3′)‐O‐(4‐benzoylbenzoyl) adenosine 5′‐triphosphate in SOD1-G93A mice (for seven days just before the onset of pathological neuromuscular features) improves morphology of neuromuscular junctions, metabolism of myofibers, proliferation/differentiation of satellite cells, overall ameliorating denervation atrophy in ALS skeletal muscles (Fabbrizio et al., 2020).

**Figure 1:**
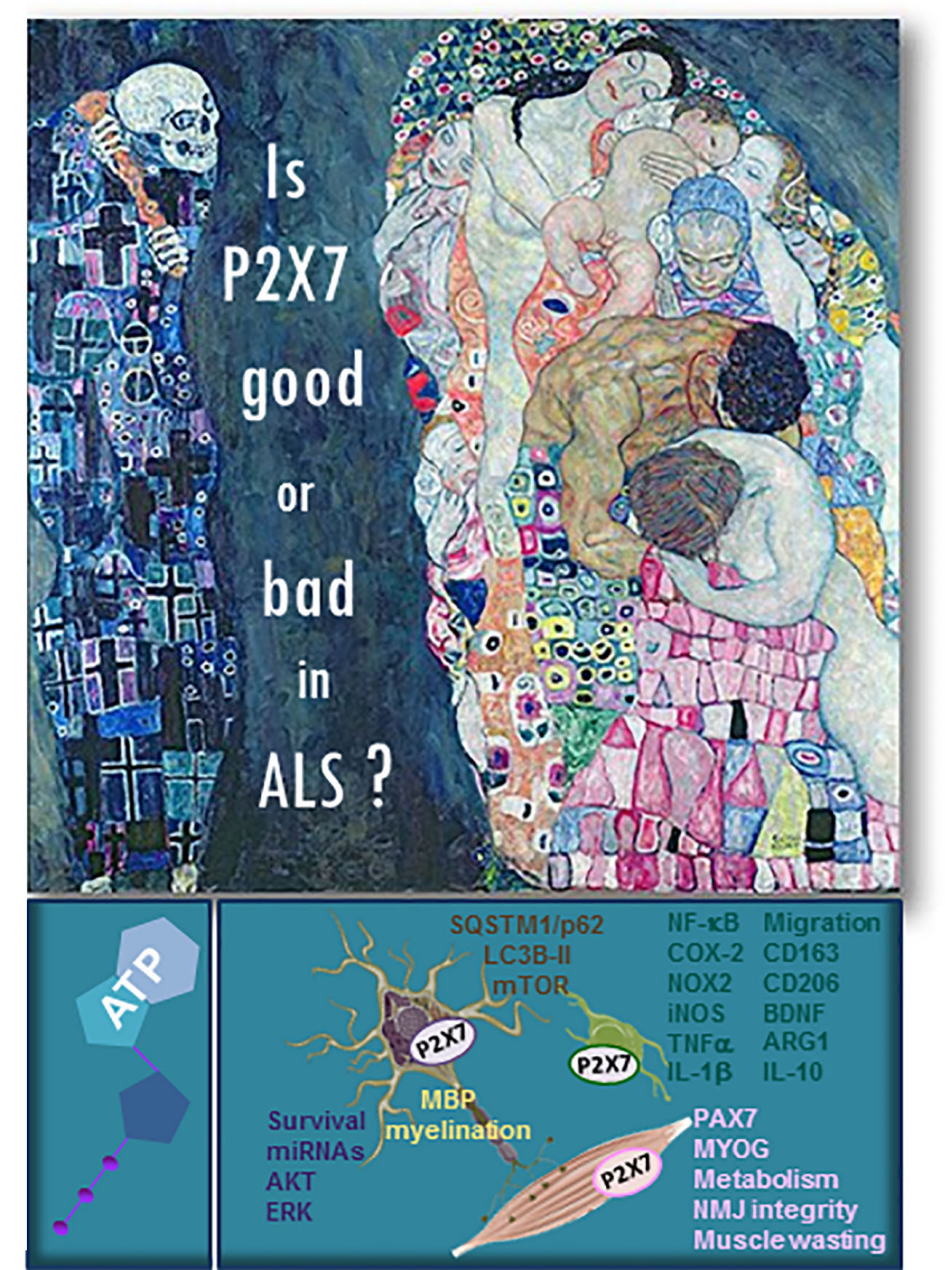
Is P2X7 good or bad in ALS? The “Death and Life” painting by Gustav Klimt (1910/15) well exemplifies the duality of purinergic P2X7 receptor in ALS. The main mechanisms and pathways involving P2X7 in ALS motor neurons (in blue color), oligodendrocytes (in yellow), microglia (in green), and muscle (in pink) pathology are listed in the figure. In brown are written the pathways shared between neurons and microglia.

These two independent sets of results confirm the duality of P2X7 in ALS (**Figure 1**) and the importance of the environmental niche as a combination of peripheral *versus* central clues that drive motor neuron and neuromuscular impairment, paralysis, and finally death in ALS.

## Beneficial and Detrimental Effects

The complex behavior of P2X7 in the ALS environment is further proven by some apparently incongruous results obtained by genetic deletion of P2X7 in SOD1-G93A mice compared to pharmacological blockade of the receptor. In P2X7−/−/SOD1-G93A mice, the insurgence of ALS symptoms is anticipated, and the disease progression is worsened. Exacerbation of pro-inflammatory gliosis and aggravated motor neuron death are also evident in these P2X7−/−/mice. The possible participation of P2X7 activity to some beneficial function, at least in some disease phases, is the most reasonable explanation (Apolloni et al., 2013a). On the other hand, pharmacological inhibition of the receptor in SOD1-G93A mice by the antagonist Brilliant Blue G generates different degrees of therapeutic efficacy on motor impairment, body weight loss, survival, and neurodegeneration/neuroinflammation in the spinal cord (Cervetto et al., 2013; Apolloni et al., 2014; Bartlett et al., 2017; Sluyter et al., 2017). This clearly indicates that P2X7 activity is undoubtedly deleterious after disease onset and must be attenuated to alleviate ALS symptoms in mice. However, new generation P2X7 antagonists that are more potent and specific than Brilliant Blue G as A804598 and JNJ-47965567 have failed under the same regard (Fabbrizio et al., 2017; Ly et al., 2020). There is no need to say that further research about optimized dosing regimens based on P2X7 pharmacokinetic data and P2X7 druggability becomes mandatory for explaining the duality of P2X7 in ALS and the molecular pathways that directly involve this receptor in the disease.

## Early and Late, Short and Prolonged Actions

We have described so far that a strong impact on ALS pathology is contributed by what happens in the local CNS compartment of neurons and glia *versus* the peripheral compartment of immune cells and muscle and by the mode (beneficial or detrimental) in which the P2X7 transduction mechanisms are delivered. Now, we claim that also the temporal component is crucial for determining how P2X7 behaves in ALS. For instance, P2X7 is known to play a complex role in regulating autophagy and autophagy-based secretion of IL-1*β* from the microglia (Takenouchi et al., 2009). Moreover, P2X7 acts as a positive autophagy regulator in monocytes and macrophages during mycobacterial infections (Biswas et al., 2008) and in dystrophic muscles (Young et al., 2015). In the ALS context, we know that a short P2X7 activation sustains a positive flux of autophagy by upregulating LC3B-II protein *via* the mTOR pathway and downregulating SQSTM1/p62 levels in SOD1-G93A primary microglia, concurrently with induction of M2 anti-inflammatory markers. Conversely, a prolonged stimulation of P2X7 leads to reduction of the autophagic flux, with detrimental accumulation of SQSTM1/p62 protein and concomitant M1 pro-inflammatory polarization of SOD1-G93A microglia (Fabbrizio et al., 2017). We can easily correlate the P2X7-short-time-evoked stimulation of autophagy with the beneficial activation of P2X7 likely occurring during the early asymptomatic phase of ALS, precisely when the genetic ablation of the receptor becomes detrimental in SOD1-G93A mice (Apolloni et al., 2013a). Instead, persistent activation of P2X7 leading to deleterious inhibition of autophagy might resemble what occurs during the later symptomatic phase of the disease, exactly when P2X7 needs to be inhibited for contrasting ALS progression (Apolloni et al., 2014; Sluyter et al., 2017). In other words, these results confirm that the duality of the time-specific participation of P2X7 to ALS extends to autophagic other than neuroinflammatory mechanisms.

While we expect to corroborate the notion that pathological mechanisms of ALS indeed proceed through pathways in which P2X7 plays a central role, under this same perspective we must now further integrate insights from animal models and human studies, converging genomics, transcriptomics, proteomics, and metabolomics with computational biology.

## Conclusive Discussion

This opinion article has discussed some challenges and reasons for further promoting research in the field of P2X7 and ALS. We have reported that the P2X7 environmental niche combining peripheral/central clues that drive motor neuron impairment and the early/late and short/prolonged timing of beneficial *versus* detrimental P2X7 responses, becomes very important for further understanding ALS. However, conflicting data still raise questions about the origin of dominant mechanisms in ALS and their P2X7-dependent fate. Here, we have come to the conclusion that further research about P2X7, its dosing and druggability, is needed to deepen our understanding of the pathways involved in the insurgence and progression of ALS.

In doing so, we have described that the key to direct a whole new interdisciplinary field of P2X7 in ALS must be by integrating insights from humans with animal models, bridging genomics, transcriptomics, proteomics, and metabolomics with computational biology. Nevertheless, the duality of the participation of P2X7 to ALS remains an important layer to be explored for discerning and directing the pathological processes of the disease (**Figure 1**). The summation of these layers and the success of these research fields will be a paradigm of the power of interdisciplinary P2X7 research in ALS.

Although the field of P2X7 and ALS may appear mature, it is so new that many questions continue to arise, and we can look forward to exciting new developments. Designing a clinical trial, identifying targets, outcomes, and biomarkers is only a first step in the ultimate goal of translating the P2X7 knowledge into the clinic. The right answer to ALS is conceivably a multidrug strategy and/or the use of broad-spectrum molecules that, in our opinion, will surely comprise the P2X7 receptor among their targets.

## Author Contributions

CV conceived the work, drafted the manuscript, and composed the figure. SA, FL, and PF contributed in writing and editing.

## Funding

The Italian Ministry of Health has supported this work through Ricerca Corrente to CV and grant SG-2018-12366226 to PF.

## Disclaimer

This is an opinion article based on literature review. No experiments have been conducted or data collected.

## Conflict of Interest

The authors declare that this work was conducted in the absence of any commercial or financial relationships that could be construed as a potential conflict of interest.

## Abbreviations

ALS, amyotrophic lateral sclerosis; SOD1, superoxide dismutase 1.
